# Reinforcing endothelial junctions prevents microvessel permeability increase and tumor cell adhesion in microvessels *in vivo*

**DOI:** 10.1038/srep15697

**Published:** 2015-10-28

**Authors:** Bingmei M. Fu, Jinlin Yang, Bin Cai, Jie Fan, Lin Zhang, Min Zeng

**Affiliations:** 1 Department of Biomedical Engineering, The City College of the City University of New York, 160 Convent Ave, New York, NY 10031.

## Abstract

Tumor cell adhesion to the microvessel wall is a critical step during tumor metastasis. Vascular endothelial growth factor (VEGF), a secretion of tumor cells, can increase microvessel permeability and tumor cell adhesion in the microvessel. To test the hypothesis that inhibiting permeability increase can reduce tumor cell adhesion, we used *in vivo* fluorescence microscopy to measure both microvessel permeability and adhesion rates of human mammary carcinoma MDA-MB-231 cells in post-capillary venules of rat mesentery under the treatment of VEGF and a cAMP analog, 8-bromo-cAMP, which can decrease microvessel permeability. By immunostaining adherens junction proteins between endothelial cells forming the microvessel wall, we further investigated the structural mechanism by which cAMP abolishes VEGF-induced increase in microvessel permeability and tumor cell adhesion. Our results demonstrate that 1) Pretreatment of microvessels with cAMP can abolish VEGF-enhanced microvessel permeability and tumor cell adhesion; 2) Tumor cells prefer to adhere to the endothelial cell junctions instead of cell bodies; 3) VEGF increases microvessel permeability and tumor cell adhesion by compromising endothelial junctions while cAMP abolishes these effects of VEGF by reinforcing the junctions. These results suggest that strengthening the microvessel wall integrity can be a potential approach to inhibiting hematogenous tumor metastasis.

Increase in microvessel permeability is a critical step in many pathological processes including tumor metastasis^1^,^2^. Vascular endothelial growth factor (VEGF), a family of tumor angiogenic factors, has long been recognized to enhance microvascular permeability^3^,^4^,^5^,^6^,^7^ and increase tumor cell adhesion to endothelium both *in vivo* and *in vitro*, under static and flow conditions^8^,^9^,^10^. Recent advances in the understanding of the molecular mechanisms of VEGF action have led to many anti-metastatic therapies, which include antibodies to block the binding of VEGF to its cellular receptors, small-molecule chemical inhibitors of the tyrosine kinase functions of the VEGF receptors, and antisense nucleic acids to interfere with cellular production of VEGF^4^,^8^,^9^,^10^,^11^,^12^,^13^,^14^. However, anti-metastatic strategies targeting VEGF-mediated microvessel hyperpermeability by enhancing the barrier function of the microvessel wall have not been well developed^15^,^16^.

Previous studies have found that increased intracellular levels of adenosine 3′, 5′-cyclic monophosphate (cAMP) can block the inflammatory response in a variety of experimental models. Simultaneous stimulation of adenylate cyclase and phosphodiesterase inhibition to increase endothelial cAMP levels was highly effective in inhibiting permeability increases induced by H_2_O_2_ in isolated rabbit lung^17^ and in preventing capillary leakiness by lipopolysaccharide (LPS)-induced systemic inflammation in rats^18^. Previous studies on intact microvessels also confirmed that pretreatment with 8-bromo cAMP, a cAMP analog, can abolish the microvessel hyperpermeability induced by ATP^19^, platelet-activating factor (PAF)^20^, bradykinin^21^ and VEGF^22^,^23^.

The cleft between adjacent endothelial cells is widely believed to be the principal pathway for water and hydrophilic solute transport through the microvessel wall under normal physiological conditions^24^. Inside the interendothelial cleft, junctions appear as discrete sites of fusion between the outer plasma membrane of adjacent cells when visualized in ultra-thin section electron microscopy. They create an intercellular barrier for material transport across the microvessel wall and can be regulated by response to physiological and tissue-specific requirements^25^. Using cell monolayers, many *in vitro* studies have showed that cAMP induces decreased paracellular permeability by a mechanism correlated to an increase in the number of junction strands or complexity^26^,^27^,^28^,^29^,^30^,^31^,^32^. To investigate the structural mechanism by which elevated intracellular cAMP levels regulate microvessel permeability *in vivo*, Adamson *et al.*^33^ quantified the number of junction strands in the interendothelial cleft from the same frog mesenteric microvessel on which permeability was determined^33^. Their study revealed that enhancement of intracellular cAMP levels decreases microvessel permeability by inducing an increase in the number of junction strands.

The structural mechanisms which have been studied to increase microvessel permeability by VEGF, inflammatory mediators and physical stimuli involve the formation of gaps between adjacent endothelial cells in venular microvessels, vesiculo-vacuolar organelles (VVOs) pathways, transcellular pores and fenestra^34^. For transient increase in microvessel permeability induced by VEGF, Fu and Shen^5^ suggested that the most likely structural changes would be a ~2.5-fold increase in the width of the cleft between endothelial cells forming the microvessel wall and partial degradation of endothelial surface glycocalyx. By combining a mathematical model with the permeability measurement on frog mesenteric microvessels, Fu *et al.*^22^ also predicted that the inhibitory effect of cAMP on VEGF-induced microvessel hyperpermeability is to generate more junction strands in the interendothelial cleft.

Therefore, on the basis of the previous studies on VEGF and cAMP effects on the microvessel permeability, the first aim of this study was to confirm that elevation of intraendothelial cAMP levels also abolishes/attenuates the transient increase in microvascular permeability by VEGF in intact rat microvessels. To do this, the microvessel permeability (P) to various sized solutes was measured in individually perfused post-capillary venules of rat mesentery^10^,^22^ under cAMP and VEGF treatments.

Our *in vitro* static study using cultured endothelial cell monolayers demonstrated that tumor cells prefer to adhere to the junctions of endothelial cells instead of cell bodies^9^,^35^. It was found that tumor secretion VEGF disrupts endothelial junction proteins to enhance the exposure of the underlying basement membrane to promote tumor cell adhesion to the adhesion molecules (e.g. laminins) in the extracellar matrix^8^,^9^,^36^. Thus, the second aim of this study was to investigate whether or not tumor cells prefer to adhere to the junctions of endothelial cells in intact microvessels under normal blood flow conditions. Consequently, the ultimate aim was to test the hypothesis that inhibiting VEGF-enhanced microvessel permeability by cAMP can also inhibit tumor cell adhesion to the microvessel wall; and this inhibition is through reinforcing the endothelial junctions. To accomplish these aims, *in vivo* fluorescence microscopy was employed to measure adhesion rates of human mammary carcinoma MDA-MB-231 in post-capillary venules of rat mesentery under flow and under the treatment of VEGF and cAMP; silver staining technique was used to identify the endothelial borders and adherent tumor cells; fluorescence immunostaining was applied to label adherens junction proteins between endothelial cells forming the microvessel wall under VEGF and cAMP treatments.

## Results

### Effect of cAMP on VEGF-enhanced Microvessel Permeability

**Figure 1:**
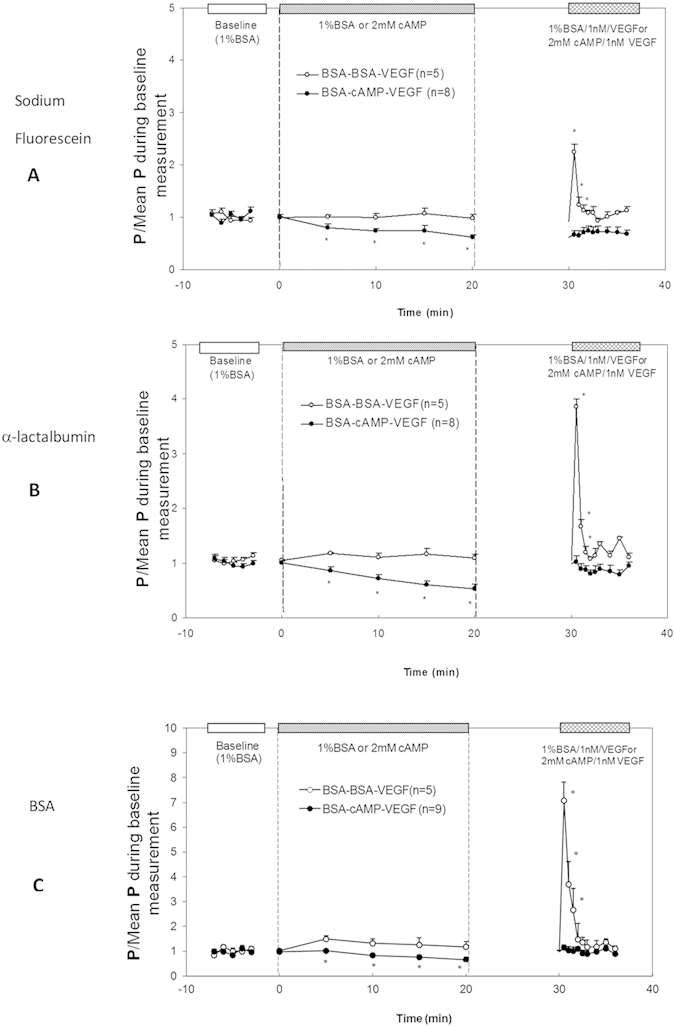
Effect of cAMP on VEGF-induced hyperpermeability in rat mesenteric microvessels for (**A**) sodium fluorescein, (**B**) α-lactalbumin and (**C**) bovine serum albumin (BSA). Mean ± SE P relative to baseline plotted as a function of time. In the matched control group (○, BSA-BSA-VEGF), baseline P was first measured with Ringer perfusate containing 1% BSA, then P was measured in a sham experiment of reperfusion with control solution for ~20 min, and finally the P was measured under the treatment of 1 nM VEGF for ~5 min. In the test group (•, BSA-cAMP-VEGF), baseline P was first measured with perfusate containing 1% BSA, then P was measured in the test experiment of reperfusion with the same solution also containing 2 mM 8-bromo-cAMP for ~20 min, and finally the P was measured under the treatment of 1 nM VEGF and 2 mM 8-bromo-cAMP for ~5 min. *p < 0.05 compared with the baseline.

Perfusion with solutions containing 2 mM 8-bromo-cAMP elicited a consistent decrease in apparent permeability P for all three molecules, and abolished the transient increase induced by 1 nM VEGF. Figure 1 summarizes the results from a series of individual P measurements in post-capillary venules of rat mesentery. Figure 1A–C demonstrate microvessel permeability to a small solute sodium fluorescein (MW = 376, P^sodium fluorescein^), to an intermediate-sized solute α-lactalbumin (MW = 14,176, P^α-lactalbumin^) and to a large solute BSA (MW = 67,000, P^BSA^), respectively. In the matched control group (shown in ○), after control and 20 min sham experiments, P^sodium fluorescein^ (Mean ± SE) in 5 vessels, measured at the peak of the response (~30 sec) to 1 nM VEGF, was 6.4 ± 0.74 × 10^−5^ cm/s, compared with a baseline P of 3.0 ± 0.24 × 10^−5^ cm/s, representing a 2.2 ± 0.13-fold increase in the same vessel (p < 0.01). In the test group (shown in •), after 20 min pretreatment of 2 mM cAMP, P^sodium fluorescein^ decreased from 3.4 ± 0.24 × 10^−5^ cm/s to 2.1 ± 0.18 × 10^−5^ cm/s (n = 8, p < 0.01), representing a mean ratio of 0.64 ± 0.051 to the baseline. At ~30 s after perfusion with 1 nM VEGF/2 mM cAMP following 20 min cAMP pretreatment, P^sodium fluorescein^ was 3.1 ± 0.18 × 10^−5^ cm/s; the mean ratio to the baseline was 0.92 ± 0.08 (p = 0.54). Pretreatment of 2 mM cAMP completely abolished the VEGF-induced transient permeability increase.

Figure 1B,C show the results for P^α-lactalbumin^ and P^BSA^, respectively. In the matched control group (shown in ○), after control and 20 min sham experiments, P^α-lactalbumin^ and P^BSA^, measured at the peak of the response (~30 sec) to 1 nM VEGF, was 2.5 ± 0.54 and 0.37 ± 0.051 × 10^−5^ cm/s, compared with baseline P of 0.63 ± 0.051 and 0.052 ± 0.0041 × 10^−5^ cm/s, representing 3.9 ± 0.6-fold and 7.1 ± 0.8-fold increases, respectively (n = 5, p < 0.01). In the test group (shown in •), after 20 min treatment of 2 mM cAMP, P^α-lactalbumin^ decreased to 0.55 ± 0.073 (n = 8, p < 0.05) and P^BSA^ to 0.65 ± 0.098 (n = 9, p < 0.05) of their baseline values, respectively. Same as for a small solute, pretreatment of 2 mM cAMP completely abolished the VEGF-induced transient P increase for both intermediate and large sized solutes. At ~30 sec after perfusion with 1 nM VEGF/2 mM cAMP, P^α-lactalbumin^ and P^BSA^ were 1.02 ± 0.11-fold (p = 0.86) and 1.17 ± 0.11-fold (p = 0.24) that of their baseline values, respectively. Table 1 summarized the permeability data under the control, cAMP and cAMP/VEGF treatment for all three solutes. In another group of 8 vessels, 20 min pretreatment of 4 mM cAMP reduced P^BSA^ to 0.50 ± 0.05-fold (p = 0.03) that of its baseline value, which was not significantly different from that by 2 mM cAMP pretreatment (p = 0.17). However, following 20 min 4 mM cAMP pretreatment, at ~30 s after perfusion with 1 nM VEGF/4 mM cAMP, P^BSA^ only returned to 0.77 ± 0.02-fold (p < 0.01) that of its baseline.

### Effect of cAMP on VEGF-enhanced Tumor Cell Adhesion to the Microvessel Wall

**Figure 2:**
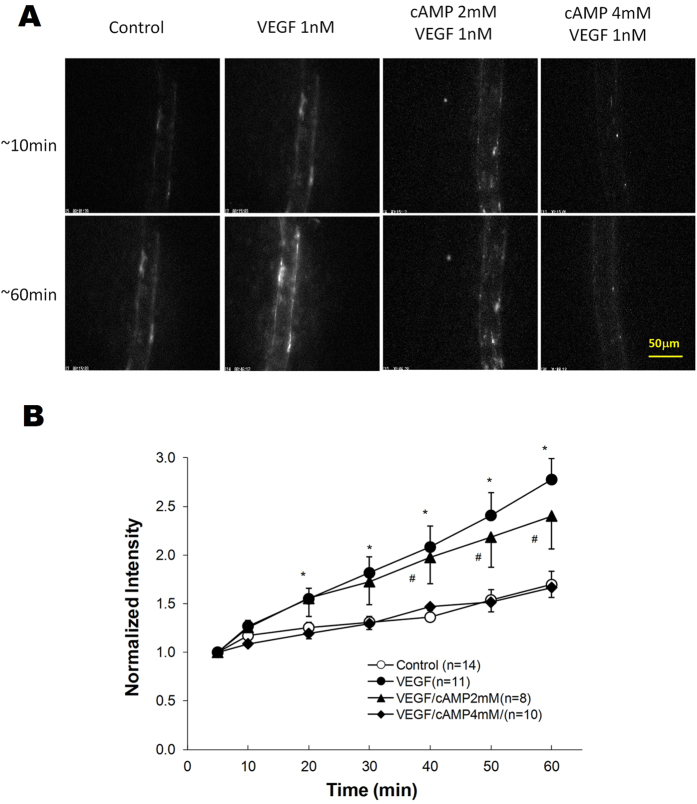
(**A**) Photomicrographs showing *in vivo* MDA-MB-231 cell adhesion to individually perfused microvessels in a control condition with 1%BSA-Ringer solution perfusate (first column), during treatment with 1 nM VEGF in the perfusate (second column), following 20 min pretreatment of the vessel with 2 mM cAMP and then during treatment with 1 nM VEGF/2 mM cAMP in the perfusate (third column) and following 20 min pretreatment of the vessel with 4 mM cAMP and then during treatment with 1 nM VEGF/4 mM cAMP in the perfusate (fourth column). The top row is for those after ~10 min perfusion and the bottom row for those after ~60 min perfusion. The perfusion velocity was ~1 mm/s, which is the normal blood flow velocity in mesenteric post-capillary venules. (**B**) Adhesion of MDA-MB-231 cells as a function of time. Adhesion is shown in control conditions with 1% BSA-Ringer perfusate (○), during treatment with 1 nM VEGF in the perfusate (•), following 20 min pretreatment of the vessel with 2 mM (▲) and 4 mM (♦) cAMP. *p < 0.001, #p < 0.05 compared with the control conditions. Values are means ± SE.

Adhesions of MDA-MB-231 cells in representative microvessels under various conditions are shown in Fig. 2A. Top panel images were taken at ~10 min and bottom images at ~60 min after perfusing tumor cells into individual microvessels. The perfusion velocity was ~1 mm/s, a mean blood flow velocity in this type of microvessels. Bright spots in these images represent adherent fluorescently-labeled tumor cells.

Figure 2B presents the time course of the amount of adherent tumor cells as indicated by fluorescence intensity in a vessel segment, under control (shown in ○), 1 nM VEGF treatment (•), pretreatment with 2 mM cAMP and then 2 mM cAMP/1 nM VEGF treatment (▲) and pretreatment with 4 mM cAMP and then 4 mM cAMP/1 nM VEGF treatment (♦). Linear regression of data for intensity (cell adhesion amount) vs. time revealed that cell adhesion increased almost linearly with time from its baseline value starting at ~5 min under control and under all treatments (R^2^ > 0.96). The cell adhesion rate (the slope of the intensity vs. time curve) under 1 nM VEGF treatment was 2.8-fold that of the control value (p = 0.03), 20 min pretreatment with 2 mM cAMP insignificantly reduced the adhesion rate to 2.5-fold that of the control value (p = 0.81 compared with VEGF alone). However, 20 min pretreatment with 4 mM cAMP completely abolished the VEGF-enhanced tumor cell adhesion (p = 0.88 compared with control).

### Preferential Location of Tumor Cell Adhesion to the Microvessel Wall

**Figure 3:**
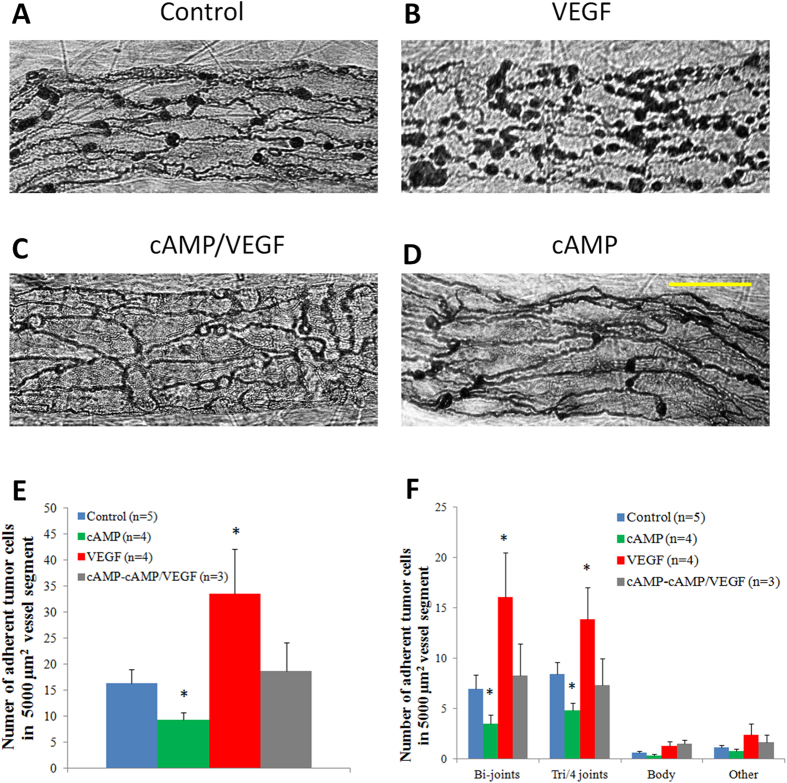
Photomicrographs showing endothelial cell boundaries and adherent MDA-MB-231 cells after 60 min perfusion from segments of individually perfused post-capillary venules in a control condition with 1% BSA-Ringer solution perfusate (A), during treatment with 1 nM VEGF in the perfusate (B), following 20 min pretreatment of the vessel with 4 mM cAMP and then during treatment with 4 mM cAMP/1 nM VEGF (C), and following 20 min pretreatment of the vessel with 4 mM cAMP and then during treatment with 4 mM cAMP alone (D). Endothelial cell borders were outlined with silver nitrate. The images were taken with a Nikon Fluor 40×/NA1.3, oil objective. Scale bar is 30 μm. Comparison of adhesion of MDA-MB-231 tumor cells to the microvessel wall in a control condition with 1% BSA-Ringer solution perfusate (in 5 vessels, blue bar), during treatment with 1 nM VEGF in the perfusate (in 4 vessels, red bar), following 20 min pretreatment of the vessel with 4 mM cAMP and then during treatment with 4 mM cAMP/1 nM VEGF (in 3 vessels, grey bar), and following 20 min pretreatment of the vessel with 4 mM cAMP and then during treatment with 4 mM cAMP alone (in 4 vessels, green bar). (**E**) Number of total adherent tumor cells in vessel segments (**F**) Number of adherent tumor cells at junctions between two (Bi-joints), three and four (Tri/4 joints) endothelial cells, at endothelial cell bodies (Body) and at uncertain locations (Other) in vessel segments. *p < 0.001, compared with controls. Values are means ± SE.

The above results showed that VEGF increased microvessel permeability and tumor cell adhesion to the microvessel wall and 4 mM cAMP could completely abolished these effects of VEGF. However, it is unclear how this was accomplished. To investigate the structural mechanism of inhibiting effects of cAMP on microvessel permeability and tumor cell adhesion, we first identified the adhesion location of tumor cells to the microvessel wall under flow. The endothelial cell boundaries were labeled by silver staining. Figure 3A–D demonstrate the photomicrographs of adherent MDA-MB-231 cells to the endothelia forming the microvessel wall after 60 min perfusion under control, treatment of VEGF, 20 min pretreatment of 4 mM cAMP and then cAMP/VEGF, and 20 min pretreatment of 4 mM cAMP and then cAMP, respectively. We grouped the locations of the adherent tumor cells into four categories: at the joint of two endothelial cells, at the joint of three (and four) cells, at the cell body and at uncertain locations such as at the edge of the vessel segment. Figure 3E compares the total number of adherent tumor cells per 5000 μm^2^ of the mid-plane area in a vessel segment (~100–150 μm long) under these conditions. After 20 min pretreatment of 4 mM cAMP, adherent tumor cells decreased from a control of 16.4 ± 2.6 to 9.3 ± 1.5 cells per 5000 μm^2^ (p < 0.001). 1 nM VEGF increased adherent tumor cells to 33.6 ± 8.7 cells per 5000 μm^2^ (p < 0.001). This increase can be inhibited by 20 min pretreatment of 4 mM cAMP, under which the number of adherent tumor cells was 18.7 ± 5.4 cells per 5000 μm^2^, comparable to the control (p = 0.51). Out of these adherent tumor cells, Fig. 3F shows that the percentages of adherent tumor cells are 42.2%, 37.1%, 47.9% and 46.3% to the bi-joints of endothelial cells; 51.3%, 51.5%, 41.2% and 42.1%, to the tri- and four joints; 2.7%, 3.4%, 3.9% and 4.9% to the cell body; and 3.8%, 8.0%, 7.0% and 6.7%, to the uncertain place, respectively, under control, cAMP, VEGF and cAMP-cAMP/VEGF treatments. More than 88% of the adherent tumor cells are at the endothelial junctions and less than 5% are on the cell bodies for all the cases. Almost all the effects of VEGF and cAMP on tumor cell adhesion are reflected from the amount of adherent tumor cells on endothelial junctions.

### Effects of cAMP and VEGF on Endothelial Junction Proteins

**Figure 4:**
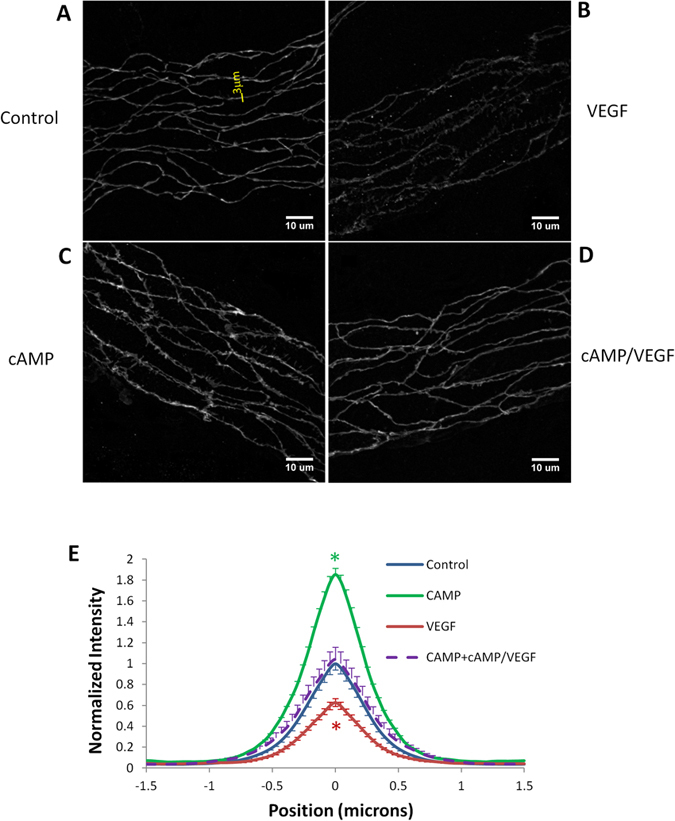
Vascular endothelial (VE)-cadherin distribution in perfused microvessels. (**A**) In control vessels with 1% BSA-Ringer solution perfusate, the VE-cadherin label appeared as a continuous and smooth band; (**B**) in VEGF-treated vessels near the peak of the permeability response, the VE-cadherin label was nonuniform and discontinuous in some regions; (**C**) in cAMP-treated vessels, the VE-cadherin label was continuous, appeared in spikes and more intense compared with that in control vessels; (**D**) in vessels pretreated with cAMP, the VEGF treatment did not induce visible changes in the VE-cadherin label compared with control. (**E**) Normalized intensity profiles of VE-cadherin label distribution (along a 3 μm perpendicular line to the cell-cell junction, shown in **A**). For each treatment, there was a control vessel prepared and imaged during the same time. The peak intensity of the VE-cadherin label from the control vessel was used to normalized that in the treated vessel. n = 80 measured profiles from about 20 cell pairs for each vessel. n = 640 for the control group, n = 160 for the cAMP group, n = 240 for the VEGF group as well as the cAMP/VEGF group. *p < 0.05 compared with controls. Values are means ± SE.

The above observation that cAMP reduced VEGF-enhanced microvessel permeability and tumor cell adhesion to the joints of endothelial cells forming the microvessel wall led us to the hypothesis that VEGF increases microvessel permeability by disrupting the integrity of the intercellular junctions while cAMP can reinforce them. To test this hypothesis, we quantified the endothelial adherens junction protein, vascular endothelial (VE)-cadherin, under control and cAMP/VEGF treatments. Figure 4A–D demonstrate the images for immunofluorescence-stained VE-cadherins at microvessel walls under control, after ~30 sec treatment of 1 nM VEGF, after 20 min treatment of 4 mM cAMP, and pretreatment of 4 mM cAMP for 20 min and ~30 sec treatment of 1 nM VEGF/4 mM cAMP, respectively. Figure 4E shows the normalized intensity profiles of VE-cadherin distribution (along a 3 μm perpendicular line to the cell-cell junction, shown in Fig. 4A). For each treatment, there was a control vessel prepared and imaged during the same time and the peak intensity value for the VE-cadherin labeling under the control was used to normalize the values under the treatment. Based on the kurtosis analysis, after 20 min treatment of 4 mM cAMP (green line), the intensity value of VE-cadherin labeling near the inter-endothelial joints is significantly higher than that of the control (blue line) (p = 0.04), the VE-cadherin labeling under acute treatment of 1 nM VEGF (red line) has a lower intensity value than that of the control (p = 0.006), while the VE-cadherin intensity value is comparable to the control level under 20 min pretreatment of 4 mM cAMP and acute treatment of 1 nM VEGF (purple line) (p = 0.15). These results conform to the hypothesis that cAMP inhibits the VEGF-enhanced microvessel permeability and tumor cell adhesion by reinforcing endothelial junctions.

## Discussion

cAMP is one of the few signaling molecules that in general improve endothelial barrier function. Elevation of endothelial cAMP appeared effective in reducing the permeability of endothelial cells both *in vitro* and *in vivo*^15^,^17^,^26^,^27^,^29^,^37^,^38^. The effect is fast and occurs both under basal conditions and stimulated states such as in ischemia-reperfusion injury^39^, and after exposure to inflammatory agents, e.g., histamine, thrombin, ATP, PAF, H_2_O_2_, bradykinin^17^,^19^,^20^,^21^,^26^,^40^,^41^. Its efficacy is independent of whether cAMP is elevated by activation of adenylate cyclase or by the inhibition of cAMP-degrading phospodiesterases^26^,^28^,^31^,^38^, which broadens possible therapeutic approaches^15^. To search for the barrier-improving agents that can protest against microvascular hyperpermeability during tumor growth and metastasis, the first aim of our study was to test the hypothesis that elevation of the intraendothelial cAMP levels also abolishes/attenuates the transient increase in microvascular permeability by a tumor angiogenic factor, VEGF, in intact microvessels. Our results shown in Fig. 1 for the microvessel permeability to small, intermediate and large sized solutes have conformed to our hypothesis. Figure 1A–C also show that the lines for permeability under cAMP/VEGF treatments are slightly lower than those under VEGF treatment in the later time after the transient increase by VEGF. This implies the stabilizing effect of cAMP on permeability.

Recent review by Sayner^38^ presented the current themes of cAMP signaling pathways that regulate endothelial barrier integrity. Endothelial barrier integrity is maintained by transmembrane cell adhesion molecules that anchor adjacent cells to each other as well as anchoring cells into the underlying matrix. To perform this adhesive function, adhesion molecules are tethered into the cortical actin cytoskeleton by adaptor molecules, such as α-, β-, and γ-catenin, plakoglobin, and α-actinin, which, together with p120, form junctional complexes^42^,^43^. Adherens junctions formed by VE-cadherins are the major junctional barrier between adjacent endothelial cells lining the microvessel wall^24^,^44^. Tight junctions comprised of ZO-1,-2,-3, together with occludins and claudins, were also identified in endothelial cells^25^,^45^,^46^. Adherens junctions, along with tight junctions, create a regulated paracellular barrier to the movement of water and solutes between endothelial cells^47^,^48^ and determine the microvessel permeability to water and hydrophilic solutes. Many previous studies have found that cAMP caused activation of Rac1 via both protein kinase A and Epac-triggered activation of Rap1, in order to modulate endothelial barrier integrity of microvessels by reinforcing adherens and tight junctions^21^,^49^,^50^,^51^. Our immuno-fluorescence staining for VE-cadherins under cAMP treatment is consistent with this observation (Fig. 4C,E).

Via activation of endothelial receptors, followed by fluxes of calcium ions, nucleotides, phospholipids, and ionic second messengers, VEGF increases vascular permeability by inducing cytoskeleton tension and disrupting endothelial junction barriers^8^,^9^,^52^. Recent review by Bates^53^ further reported that VEGF activation of endothelial cells results in phosphorylation and disassembly of VE-cadherin. VEGF mediates a dephosphorylation of VE-cadherin, which disassociates from src and Csk, a negative regulator of src, thus enabling src to be activated. Results shown in Fig. 4B,E for the reduced VE-cadherins at the endothelial junctions confirm that VEGF compromised endothelial junction integrity in microvessel walls. Although other structural changes induced by VEGF were also observed in different types of blood vessels and under various conditions, such as vesiculo-vacuolar organelles (VVOs) pathways, transcellular pores and fenestra^34^, the transient increase in the width of the interendothelial cleft predicted by Fu and Shen^5^ provided an alternative. By forming new junction strands with pretreatment of 8-bromo-cAMP, the reinforced barriers in the cleft may diminish the increase in its width by VEGF and thus prevent the increase in microvessel permeability^22^. That the pretreatment of cAMP led the VE-cadherin levels return to the control levels after VEGF treatment (Fig. 4D,E) is consistent with the prediction from Fu *et al.*^22^.

Tumor cell metastasis through blood circulation is a complex process and is one of the great challenges in cancer research as metastatic spread is responsible for ~90% of cancer-related mortality^54^. Tumor cell adhesion to the microvessel wall is one critical step in metastatic spread. Previous study on intact microvessels indicated that adhesion of tumor cells to microvessel walls degrades the endothelial surface glycocalyx, another important structural component maintaining endothelial integrity^35^,^55^. So far, there is no direct experimental investigation on the effect of either VEGF or cAMP on the endothelial surface glycocalyx. But VEGF is likely to degrade this surface glycocalyx to expose the adhesion molecules of endothelium and those in the basement membrane surrounding the microvessel. Fan *et al.*^9^ in an *in vitro* study revealed that VEGF disrupts the tight and adherens junctions of endothelial monolayers, enabling the exposure of the underlying basement membrane and increasing the binding of adhesion molecules on the tumor cells to their ligands (e.g. laminins) in the extracellular matrix. That almost all the effects of VEGF and cAMP on tumor cell adhesion are reflected from the amount of adherent tumor cells on the endothelial junctions (Fig. 3) suggests that what was observed in Fan *et al.*^9^ in their *in vitro* study is also valid in intact microvessels under normal blood flow conditions.

To determine whether tumor cells prefer to adhere at the endothelial junctions rather than randomly adhere on the microvessel wall, using the same approach as in^9^,^37^,^56^, we first measured the length of endothelial junctions per unit area for the microvessel shown in Fig. 3, the averaged value of which is 104.2 ± 7.4 mm/mm^2^ (from 40 vessel segments). This junction length is then expanded by half of the contact length (diameter) of an adherent tumor cell, 3 μm; the band area is thus 31.3% ± 2.2% of the total area. If tumor cells adhere randomly to the microvessel wall, the percentage of the adherent tumor cells to the junctions should be less than 37.9%, three standard deviation larger than the mean value. However, under control and all the treatments, the percentage of adherent tumor cells to the junctions (bi-, tri- and four endothelial cell joints) is over 88%, much greater than 37.9%, indicating a preferential adhesion rather than a random process.

As shown in Fig. 2, pretreatment with 2 mM cAMP could not diminish VEGF-enhanced tumor cell adhesion although it completely abolished VEGF-enhanced microvessel permeability (Fig. 1). However, pretreatment with 4 mM cAMP could abolish both VEGF-enhanced effects, and further reduced microvessel permeability to that lower than the control value after VEGF/cAMP treatment. Based on the predictions from a mathematical model for the interendothelial cleft with single and double junction strands^22^, we found that because there were additional junction strands formed with pretreatment of 2 mM cAMP, the microvessel permeability could go back to the control due to a more tortuous pathway for the movement of substances in the interendothelial cleft. However, the increased width of the cleft by VEGF could not return to its baseline, resulting in more exposed basement membrane. In contrast, pretreatment of 4 mM cAMP could reinforce better junction barriers, which protect the width of the cleft from the increase by VEGF, and consequently abolish the VEGF-enhanced tumor cell adhesion.

By using the same single microvessel perfusion method, our previous study^10^ reported that pretreatment of tumor cells with an antibody blocking VEGF or an antibody blocking a specific integrin, and pretreatment of the microvessel with VEGF receptor inhibitor, or anti-integrin extracellular matrix ligand antibody, can reduce the tumor cell adhesion under both control and the VEGF treatment. These results suggested that the cell adhesion molecules at the tumor cells and at endothelium are required for tumor cell adhesion to the microvessel wall. However, the enhanced microvessel permeability by VEGF and the reduced permeability by cAMP affect the contact opportunity of tumor cells to the endothelium and indirectly affect the adhesion. Enhanced endothelial junctions by cAMP could compensate the disrupted junctions by VEGF and abolish the increased tumor cell adhesion to the basement membrane of endothelium.

In addition to strengthening the endothelial barrier function, Bianco *et al.*^57^ discovered that 8-Chloro-cyclic AMP, another cAMP analogue, can suppress tumor growth by inhibiting angiogenic growth factor production in human breast and colorectal cancer. That cAMP-induced decrease in VEGF generation of tumor cells may also reduce tumor cell adhesion to the microvessels and inhibit tumor metastasis.

In summary, our study on intact microvessels indicates that pretreatment of microvessels with cAMP can abolish VEGF-enhanced microvessel permeability and tumor cell adhesion through reinforcing endothelial junction integrity. These results can be applied to broaden the anti-metastatic therapies by employing many widely recognized anti-inflammatory agents targeting the endothelial barriers. In addition to the structural mechanism, the signaling mechanism by which cAMP abolishes VEGF induced increase in microvessel permeability and tumor cell adhesion remains to be investigated. An understanding of this mechanism would also help us develop new anti-metastatic therapies targeting the signaling pathway of tumor cell adhesion to the microvessel wall.

## Materials and Methods

### Animal Preparation

All experiments were performed on female Sprague-Dawley rats (250–300 g, age 3–4 months), supplied by Hilltop Laboratory Animals (Scottsdale, PA, USA). The experimental methods were in accordance with the guidelines and regulations approved by the Institutional Animal Care and Use Committee at the City College of the City University of New York. The protocol number is 925. The methods used to prepare rat mesenteries, perfusate solutions and micropipettes for microperfusion experiments have been described in detail elsewhere^10^,^35^,^58^. A brief outline of the methods was given below with emphasis on the special features of the present experiments. At the end of experiments the animals were euthanized with excess anesthetic. The thorax was opened to ensure death^58^.

Rats were first anaesthetized with pentobarbital sodium given subcutaneously. The initial dosage was 65 mg/kg and additional 3 mg/dose was given as needed. After a rat was anesthetized, a midline surgical incision (2–3 cm) was made in the abdominal wall. The rat was then transferred to a tray and kept warm on a heating pad. The mesentery was gently taken out from the abdominal cavity and spread on a glass coverslip, which formed the base of the observation platform as previously described^10^,^35^. The upper surface of the mesentery was continuously superfused by a dripper with mammalian Ringer solution at 35–37 °C, which was regulated by a controlled water bath and monitored regularly by a thermometer probe. The microvessels chosen for the study were straight non-branched post-capillary venules, with diameters of 30–50 μm.

### Solutions and Reagents

Mammalian Ringer solution was used for all dissections, perfusate and superfusate^10^,^35^. In addition, the perfusate into the microvessel lumen contained bovine serum albumin (BSA) at 10 mg/ml (1% BSA-Ringer solution). All chemicals in the Ringer solution, BSA, 8-bromo-cAMP, fluorescence labeled solutes and AgNO_3_ were purchased from Sigma-Aldrich (St. Louis, MO). Cell Tracker Green were purchased from Invitrogen (Eugene, OR). VEGF (human recombinant VEGF_165_) was from Peprotech (Rocky Hill, NJ). The goat anti-bodies against VE-cadherin were purchased from Santa Cruz Biotechnology (Santa Cruz, CA) and Alexa fluor 555 conjugated donkey anti-goat secondary antibodies from Invitrogen. Sodium fluorescein was dissolved at 0.1 mg/ml, FITC-BSA at 0.75 mg/ml in 1%BSA-Ringer. α-lactalbumin was labeled with FITC (mol wt 389.4) as in^58^. The final concentration of FITC-α-lactalbumin was 0.75 mg/ml in 1% BSA-Ringer. All of the solutions described above were made at the time when the experiment was performed and were discarded at the end of the day.

### Cell Culture and Fluorescent Tagging

Human breast carcinoma (MDA-MB-231) cells were purchased from ATCC (Manassas, VA) and cultured and labeled with Cell Tracker Orange (Invitrogen, Eugene, OR) as previously described. Concentration of cell suspension was adjusted for the final perfusate ~4 million/ml in 1% BSA-Ringer solution. The cell survival rate was >95% before perfusion, and >90% after ∼1.5 h perfusion at a rate ~1 mm/s in perfusing micropipettes.

### Measurement of Apparent Microvascular Solute Permeability P

Measurement of P was taken on the individual post-capillary venules under control, pretreatment with 8-bromo-cAMP and in the perfusate with cAMP and VEGF. The detailed method using θ pipette for P measurement was previously described in^10^,^35^,^58^. Briefly, when the solution with fluorescently labeled solutes was perfused into the vessel and the vessel was exposed to a 495 nm wavelength light, the images were recorded simultaneously by a high-performance digital 12-bit CCD camera (SensiCam QE, Cooke Corp., Romulus, MI, USA) with a Super Fluor 20 × objective lens (NA = 0.75, Nikon). Then the P was determined off-line. The total fluorescence intensity (*I*) in the lumen of a straight vessel and surrounding tissue was determined by image analysis software (Intracellular Imaging Inc., Cincinnati, OH). The measuring window was 300–500 μm long and 100–200 μm wide and was set at least 100 μm from the cannulation site and from the base of the bifurcation to avoid solute contamination from the cannulation site and from the side arms. Permeability P was calculated by P = (1/ΔI_0_)(dI/dt)_0_(r/2), where ΔI_0_ was the step increase in fluorescence intensity in the measuring window when the perfused dye just filled up the vessel lumen, (dI/dt)_0_ was the initial rate of increase in fluorescence intensity after the dye filled the lumen and began to accumulate in the tissue, and r was the vessel radius.

### MDA-MB-231 Cell Adhesion in Individually Perfused Microvessels

To measure the tumor cell adhesion rate, a single straight post-capillary venule (30–50 μm diameter) was cannulated with a micropipette (~20–30 μm tip diameter, WPI Inc., Sarasota, FL) filled with 1% BSA-Ringer solution containing ~4 million cells/ml. The venule was perfused at a flow velocity of ~1 mm/s, which is a mean blood flow velocity in the post-capillary venules of rat mesentery. The perfusion flow velocity was determined by the driving pressure and was calculated from the movement of a marker tumor cell^35^. The adhesion process was recorded at ~2 frames/s in a ~5 min interval for ~60 min in each experiment. A single experiment was carried out in one microvessel per animal. Since we used 20×/NA0.75 objective lens to observe the cell adhesion, which has a depth of light collection ~100 μm^10^,^59^, the cells adhering at the top and bottom of the vessel can also be observed when we focus at the mid-plane of the vessel. Cell adhesion was represented by the total cells (the fluorescence intensity of total adherent cells) in a vessel segment (vessel diameter wide and 300–400 μm long). *In vitro* calibration experiments demonstrated that the fluorescence intensity of the fluorescently-labeled cells is a linear function of the number of cells.

### Determination of Tumor Cell Adhesion Locations by Silver Staining Boundaries of Endothelial Cells Forming Microvessel Walls

To determine the locations of adherent tumor cells on microvessel walls, at the end of 60 min perfusion of tumor cells under different treatments (3 to 5 vessels for each group), the microvessel with adherent tumor cells was recannulated, perfused with AgNO_3_ (0.2 g/100 ml) in aqueous solution for 10–20 s, and then perfused with 1% BSA-Ringer to delineate the endothelial boundaries^10^,^60^. The images of microvessels with adherent tumor cells and silver nitrate stained endothelial cell (EC) boundaries were taken by an inverted microscope system with a 40×/NA1.3 oil objective. The locations of adherent tumor cells were identified and grouped into four categories: at the joints of two ECs, at the joints of three and four ECs, at the EC bodies and others (e.g. at boundaries of vessels).

### Immuno-labeling VE-cadherin of Microvascular Endothelial Cell Junctions

In order to quantify the microvascular EC junctions under various treatments (2 to 3 vessels for each group), we used fluorescently conjugated antibodies to label the VE-cadherin of EC junctions and used confocal microscopy for the three-dimensional scanning^20^. After being perfused with the treatment solution through a cannulated micropipette, the microvessel was fixed by superfusing the tissue with ice-cold 1% paraformaldehyde for 3–5 min. The animal was sacrificed by anesthetic overdosing. The tissue (~1 cm × 1 cm) surrounding the vessel was then dissected, rinsed with ice cold PBS, permeabilized and blocked. After incubated with primary goat antibodies against VE-cadherin (1:100) for overnight at 4 °C, the free antibodies were washed away by PBS, the tissue was incubated with Alexa fluor 555 conjugated donkey anti-goat secondary antibodies (1:200) for 1 h at 4 °C. After washing away the unbounded antibodies, the tissue was mounted on a glass coverslip. A secured-seal spacer (Invitrogen, Eugene, OR) was used to surround the tissue and to make a well about 120 μm deep between two coverslips to retain the three-dimensional structure of the vessels.

### Intravital and Confocal Microscopy

A Nikon Eclipse TE2000-E inverted fluorescent microscope was used to observe the mesentery. The tissue was observed with either transmitted white light from a light pipe suspended above the preparation or with fluorescent light from an illumination system (the monochromator with a xenon lamp FSM150Xe, Bentham Instrument Ltd., UK). The monochromator can generate the light of wavelength from 200 to 700 nm. The observation of fluorescently labeled tumor cells in microvessels and measurement of microvessel solute permeability were done by a high-performance digital 12-bit CCD camera (SensiCam QE) with a Super Fluor 20× objective lens (NA = 0.75, Nikon) and recorded by the InCyt Im^TM^ imaging and analyzing system (Intracellular Imaging Inc., Cincinnati, OH, USA). The silver nitrate stained boundaries of ECs forming the microvessel wall and the adherent tumor cells were observed by a 40× oil objective lens (NA = 1.3, Nikon).

The detailed distribution of fluorescently labeled VE-cadherin of endothelial junctions were observed using 12-bit laser scanning confocal microscopy (LSCM, Zeiss LSM 510 Confocal Microscope System) with a 63×/NA1.4 objective lens^20^. The Images were separately collected from either the top (near lens) or bottom half of each vessel, forming a stack of images along the z-direction. The thickness of each image was 0.5 μm. Stacks of 15–35 images were projected onto a single plane for VE-cadherin intensity analysis. The lateral resolution was 2048 × 2048 for a vessel segment of ~89 × 89 μm. Four segments were collected for each vessel. The image stacks were analyzed with the public domain National Institutes of Health IMAGE J program. An intensity profile of the VE-cadherin label was measured perpendicularly to the EC border at the randomly selected locations for 5 EC pairs in each segment. Four readings along each border and total 80 readings for each vessel were obtained. For each treatment vessel, there was a control vessel collected at the same time. The mean maximum intensity of the VE-cadherin label in the control vessel was used to normalize the intensity profile for the treatment and control vessels.

### Experimental Protocol

To test the role of cAMP on VEGF-induced microvessel hyperpermeability, for each test solute, after making several control measurements when the washout side was filled with 1% BSA-Ringer and the dye side was filled with the same perfusate to which the test solute was added, we replaced the pipette by a new pipette with both washout and test sides containing 2 mM 8-bromo-cAMP, which concentration was consistent with that used in^37^,^22^. After ~20 min pretreatment of 2 mM 8-bromo-cAMP, we finally replaced the pipette by another new pipette with both washout and test sides that contained 8-bromo-cAMP (2 mM) and 1 nM VEGF, which concentration was consistent with that used in^22^,^58^. P was measured every 15–30 sec including both dye (5–15 sec) and washout perfusion (10–15 sec). The alternating perfusion of dye and washout solutions lasted ~5 min. The protocol for the matched control groups was the same as for the test groups except using 1% BSA Ringer perfusates without 8-bromo-cAMP.

To test the role of cAMP on VEGF-induced tumor cell adhesion, we pretreated the vessel with 2 mM or 4 mM cAMP for 20 min before perfusing the vessel with tumor cells in 2 mM or 4 mM cAMP and 1 nM VEGF in 1% BSA Ringer. We chose 1 nM VEGF because 1) it represents the secretion level of tumor cells observed after prolonged incubation; 2) it may reflect a local VEGF level in microvasculature near a solid tumor; and 3) it was shown to be an optimal dose that significantly increased tumor cell adhesion and endothelial permeability both *in vitro* and *in vivo*^8^,^10^. 2 mM cAMP was used because it can significantly decrease microvessel permeability^22^,^37^.

### Analysis and Statistics

P measurements during the control period in a vessel were averaged to establish a single value for the baseline P. This value was then used as a reference for all subsequent measurements on that vessel. Because of the difference in the cell concentration, fluorescence labeling and vessel size for different experiments, in the cell adhesion measurement we defined a base intensity I_0_ for each vessel, which was an averaged value of 3 measurements in the first 5 min of cell perfusion. The time course of cell adhesion I(t) was normalized as 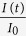. To present adhesion data at a specific time after 5 min, individual measurements were grouped with 5 min intervals at 10 min (6–10 min), 15 min (11–15 min), etc. Both results for permeability and cell adhesion are presented in mean ± SE unless specified otherwise.

Statistical significance of the treatment over time was tested with a nonparametric Wilcoxon signed-rank test applied to the averaged adhesion data. Mann-Whitney’s U test was applied to between-group data to test for adhesion differences at specific times. Significance was assumed for probability levels p < 5%.

## Additional Information

**How to cite this article**: Fu, B. M. *et al.* Reinforcing endothelial junctions prevents microvessel permeability increase and tumor cell adhesion in microvessels *in vivo*. *Sci. Rep.* **5**, 15697; doi: 10.1038/srep15697 (2015).

## Figures and Tables

**Table 1:** Control and test values of apparent solute permeability induced by 2 mM 8-bromo-cAMP (after ~20 min treatment) and 2 mM 8-bromo-cAMP/1nMVEGF (after ~30 sec treatment) in the same vessels of rat mesentery.

Solute	n	Baseline (x 10^−5^ cm/s)	cAMP (x 10^−5^ cm/s)	Ratio	cAMP/VEGF (x 10^−5^ cm/s)	Ratio
Sodium fluorescein	8	3.4 ± 0.23	2.1 ± 0.18	0.64 ± 0.051	3.1 ± 0.18	0.92 ± 0.08
α-lactalbumin	8	0.60 ± 0.04	0.38 ± 0.035	0.55 ± 0.073	0.65 ± 0.09	1.02 ± 0.11
BSA	9	0.052 ± 0.0047	0.034 ± 0.0049	0.66 ± 0.098	0.060 ± 0.0078	1.17 ± 0.11

Values are means ± SE; n, number of vessels. VEGF, vascular endothelial growth factor. Ratio is that relative to the baseline value.
